# Beyond Cutting: CRISPR-Driven Synthetic Biology Toolkit for Next-Generation Microalgal Metabolic Engineering

**DOI:** 10.3390/ijms26157470

**Published:** 2025-08-02

**Authors:** Limin Yang, Qian Lu

**Affiliations:** 1 School of Life Sciences, Jiangsu University, Zhenjiang 212100, China; yanglm@ujs.edu.cn; 2 School of Grain Science and Technology, Jiangsu University of Science and Technology, Zhenjiang 212100, China

**Keywords:** CRISPR, microalgal engineering, synthetic biology, metabolic engineering, biomanufacturing

## Abstract

Microalgae, with their unparalleled capabilities for sunlight-driven growth, CO_2_ fixation, and synthesis of diverse high-value compounds, represent sustainable cell factories for a circular bioeconomy. However, industrial deployment has been hindered by biological constraints and the inadequacy of conventional genetic tools. The advent of CRISPR-Cas systems initially provided precise gene editing via targeted DNA cleavage. This review argues that the true transformative potential lies in moving decisively beyond cutting to harness CRISPR as a versatile synthetic biology “Swiss Army Knife”. We synthesize the rapid evolution of CRISPR-derived tools—including transcriptional modulators (CRISPRa/i), epigenome editors, base/prime editors, multiplexed systems, and biosensor-integrated logic gates—and their revolutionary applications in microalgal engineering. These tools enable tunable gene expression, stable epigenetic reprogramming, DSB-free nucleotide-level precision editing, coordinated rewiring of complex metabolic networks, and dynamic, autonomous control in response to environmental cues. We critically evaluate their deployment to enhance photosynthesis, boost lipid/biofuel production, engineer high-value compound pathways (carotenoids, PUFAs, proteins), improve stress resilience, and optimize carbon utilization. Persistent challenges—species-specific tool optimization, delivery efficiency, genetic stability, scalability, and biosafety—are analyzed, alongside emerging solutions and future directions integrating AI, automation, and multi-omics. The strategic integration of this CRISPR toolkit unlocks the potential to engineer robust, high-productivity microalgal cell factories, finally realizing their promise as sustainable platforms for next-generation biomanufacturing.

## 1. Introduction

Microalgae represent a cornerstone of sustainable biomanufacturing, offering transformative solutions to global challenges in energy, nutrition, and environmental stewardship [1]. Their unparalleled advantages include rapid growth powered by sunlight and CO_2_, superior carbon fixation capabilities, and the innate capacity to synthesize diverse high-value compounds—from nutraceuticals (e.g., omega-3 fatty acids and carotenoids) and biofuels to therapeutic proteins and commodity chemicals [2,3]. These attributes position microalgae as versatile, self-sustaining cell factories poised to drive a circular bioeconomy [4].

Despite this immense promise, industrial-scale deployment remains hampered by persistent bottlenecks. Suboptimal biomass productivity, vulnerability to environmental fluctuations and pathogens, and insufficient target compound titers frequently fall below economic viability thresholds [5]. These limitations stem from inherent biological constraints and the inadequacy of conventional metabolic engineering tools. Traditional approaches, such as random mutagenesis or low-efficiency homologous recombination, lack precision, suffer from poor throughput, and are ill-suited for orchestrating complex genetic rewiring [6,7]. Consequently, efforts to enhance robustness, productivity, or novel pathway integration progress slowly and often unpredictably.

The advent of CRISPR-Cas systems marked a pivotal breakthrough, enabling targeted gene editing in microalgae with unprecedented precision [6,8]. Early CRISPR applications—primarily focused on gene knockouts and knock-ins via programmable nucleases (e.g., Cas9/Cas12)—addressed initial genetic access barriers [9,10,11]. However, merely “cutting” DNA is fundamentally insufficient to overcome the multifaceted challenges of next-generation metabolic engineering. Optimizing traits like lipid overproduction, stress resilience, or complex metabolic flux rerouting demands nuanced, multi-layered interventions: tunable gene expression control, epigenetic reprogramming, base-level corrections, and dynamic regulation across multi-gene circuits.

**Figure 1:**
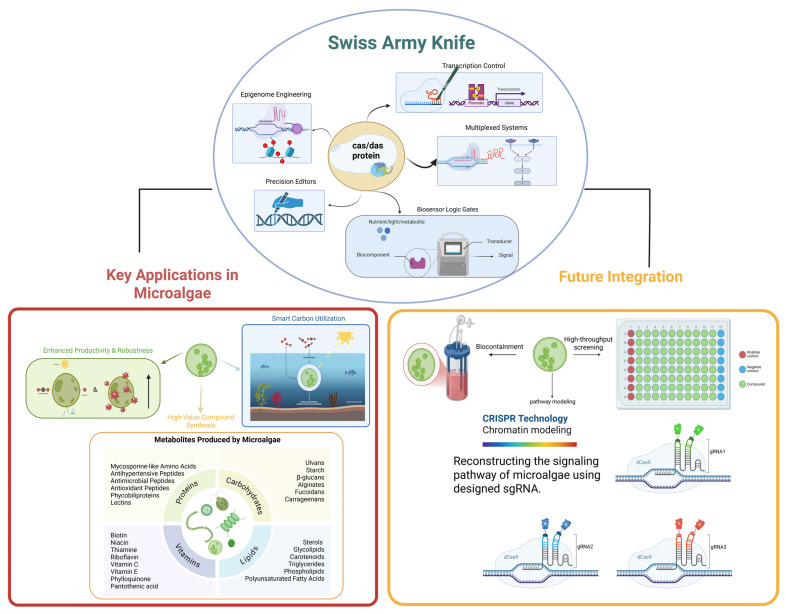
CRISPR-driven synthetic biology toolkit for microalgal metabolic engineering. This diagram illustrates a comprehensive CRISPR-based toolkit tailored for microalgal metabolic engineering. At the core are dead CRISPR proteins (dCas9/dCas12), serving as a versatile “Swiss Army Knife” with five modular capabilities: transcriptional control (Labels: A. Transcription, B. Promoter, C. Gene) epigenome engineering for stable chromatin remodeling, precision base and prime editing without double-strand breaks, multiplexed systems for coordinated pathway rewiring, and biosensor logic gates enabling autonomous dynamic control. These tools enable enhanced productivity and robustness in photosynthesis and stress tolerance, high-value compound synthesis (lipids/biofuels, carotenoids/PUFAs, proteins), and smart carbon utilization through optimized CO_2_ fixation and flux redirection. The orange box highlights the future integration of gRNA design, automation for high-throughput screening, and biocontainment strategies. The conceptual overview diagram underscores the toolkit’s multifunctionality.

Herein lies the transformative potential of CRISPR-driven synthetic biology. By repurposing CRISPR components beyond nucleases, researchers have unlocked a versatile molecular toolkit. Catalytically deactivated Cas proteins (dCas9/dCas12) serve as programmable scaffolds for transcriptional activators/repressors (CRISPRa/i), enabling precise gene expression modulation without DNA cleavage [12]. Base editors (CBEs, ABEs) facilitate single-nucleotide conversions, while prime editors (PEs) support targeted insertions, deletions, and all base transitions without double-strand breaks. Further innovations harness CRISPR for epigenetic modifications, RNA targeting, and multiplexed genome regulation [13]. This evolution—from molecular “scissors” to a synthetic biology “Swiss Army Knife”—empowers multiplexed, tunable, and context-aware control of cellular machinery, essential for engineering microalgae with industrial-grade performance (Figure 1).

This review synthesizes the rapid progress in CRISPR-driven tool development for microalgal metabolic engineering, moving decisively beyond cutting. We critically evaluate the expanding arsenal—CRISPRa/i, base/prime editors, epigenetic modulators, and multiplexing systems—and their applications in the following areas: (1) Boosting Productivity and Robustness: Enhancing growth under scale-up conditions and resilience against stressors; (2) Elevating Compound Yields: Optimizing pathways for high-value products (e.g., biofuels, nutraceuticals); (3) Engineering Complex Pathways: Rewiring metabolism via dynamic, multi-gene circuits.

We further address persistent challenges—delivery efficiency, species-specific tool optimization, off-target effects, and scalability—and highlight emerging technologies poised to unlock the full potential of microalgae as next-generation biomanufacturing platforms. By bridging cutting-edge synthetic biology with industrial imperatives, this work aims to catalyze the development of high-performance, economically viable algal cell factories.

## 2. The CRISPR Toolkit: Core Components Beyond Cutting

The transition of CRISPR technology from a simple DNA-cleaving apparatus to a multifaceted synthetic biology platform represents a quantum leap for microalgal metabolic engineering, fulfilling the promise outlined in the Introduction [14,15,16]. Moving decisively beyond the initial paradigm of targeted double-strand breaks (DSBs), this toolkit now offers the unprecedented precision, programmability, and versatility essential for overcoming the “inherent biological constraints” and “inadequacy of conventional metabolic engineering tools” that have hampered industrial-scale microalgal deployment. This section dissects the core components enabling this revolution, focusing on their principles, adaptations for microalgae, and transformative applications, thereby building the “synthetic biology arsenal” critical for creating “industrial-grade performance” strains.

### 2.1. Foundations for Precision Editing

The bedrock of advanced CRISPR applications remains the programmable Cas effector protein and the means to deliver it efficiently into the recalcitrant microalgal cell—fundamental prerequisites for moving “beyond cutting”.

#### 2.1.1. Cas Protein Variants and Algal Adaptability

While *Streptococcus pyogenes* Cas9 (SpCas9) was the pioneer, its limitations in microalgae—large size hindering delivery, strict Protospacer Adjacent Motif (PAM) requirements (5′-NGG-3′), and significant off-target effects—have spurred diversification, directly addressing the need for species-specific optimization [8,17]. Smaller orthologs like *Francisella novicida* Cas12a (FnCas12a, ~3.7 kb) offer distinct advantages: simpler crRNAs enabling multiplexing, staggered DSBs potentially favoring specific repair pathways, and alternative PAMs (e.g., 5′-TTTV-3′). Even smaller variants like the ultra-compact CasMINI (~1.5 kb), engineered from *Prevotella* sp. P5C062 Cas12f, hold immense promise for viral vector packaging or complex cargo delivery into microalgae with notoriously small cell sizes and rigid walls, overcoming a major delivery barrier [18]. High-fidelity variants (e.g., SpCas9-HF1, eSpCas9, HypaCas9), engineered with mutations reducing non-specific DNA contacts, are crucial for minimizing off-target edits in organisms like *Chlamydomonas reinhardtii*, where error-prone repair pathways can amplify errors, ensuring the precision demanded for metabolic engineering [19]. Cas12 nucleases often exhibit lower off-target rates than Cas9 and are gaining traction in diatoms and *Nannochloropsis*. The key challenge lies in empirically determining the most efficient and specific Cas ortholog–engineered variant pair for each microalgal species, often requiring codon optimization and promoter (e.g., viral, endogenous strong constitutive/inducible) screening—a critical step for unlocking the potential in diverse algal hosts [20,21]. Furthermore, fundamental differences between prokaryotic CRISPR-Cas origins and eukaryotic cellular machinery necessitate additional adaptations. These include the following: (1) Engineering nuclear localization signals (NLSs) into Cas proteins to ensure proper nuclear entry—a requirement absent in bacteria but critical for genome access in algae with defined nuclei [22]. (2) Modifying gRNA expression systems to accommodate eukaryotic RNA processing (e.g., using RNA Pol III promoters like *U6*, tRNA promoters, or ribozyme-flanked cassettes), as native bacterial crRNA arrays may not be correctly processed [23]. (3) Accounting for chromatin state differences, where heterochromatic regions can impede Cas protein binding [24,25]. (4) Navigating fundamentally distinct DNA repair pathways, particularly the inefficiency of HDR in most microalgae compared to prokaryotic homologous recombination systems [7,8]. These eukaryotic-specific barriers compound the challenges of species-specific optimization and must be addressed for robust CRISPR tool functionality [26].This empirical optimization is particularly critical given stark differences in tool performance across species. For instance, while SpCas9 is functional in the model alga *C. reinhardtii*, its editing efficiency can be suboptimal in industrially relevant strains like *Nannochloropsis gaditana*, where Cas12a (e.g., FnCas12a, LbCas12a) often demonstrates superior performance and lower off-target rates [6,27,28]. Similarly, PAM requirements pose distinct constraints: the canonical SpCas9 NGG PAM is relatively abundant in *C. reinhardtii* genomes but less frequent in some diatoms like *Phaeodactylum tricornutum*, making Cas12a variants recognizing T-rich PAMs (e.g., TTTA, TTTV) more suitable [29,30]. Even smaller variants like CasMINI show promise but require validation across diverse microalgae with varying genome complexities and GC contents [31,32,33].

#### 2.1.2. Delivery Strategies: Overcoming the Algal Fortress

Efficient intracellular delivery of CRISPR ribonucleoproteins (RNPs), mRNA, or DNA constructs remains a primary bottleneck, compounded by diverse cell wall/membrane compositions (cellulose, silica, algaenan), polysaccharide capsules, and varying cell sizes—a fundamental hurdle highlighted in the Introduction’s call for bridging “delivery efficiency” [34]. Physical methods like electroporation (widely used in *C. reinhardtii*) and particle bombardment (biolistics) offer species-agnostic delivery but suffer from low efficiency, high cell mortality, and frequent multi-copy integration causing transgene silencing, limiting their utility for generating stable, high-performing strains [17]. Chemical methods, including PEG-mediated transformation and cationic polymer/nanoparticle complexes (e.g., chitosan, polyethyleneimine), show promise for non-model species but require extensive optimization for each algal type [8,17]. Biological vectors, particularly engineered viruses (e.g., *Chlorella* viruses) or advanced Agrobacterium-based systems adapted for algae, represent a frontier for high-efficiency, and potentially low-copy, stable delivery, crucial for industrial applications [35]. Recent innovations focus on cell wall-weakening enzymes (e.g., cellulase pretreatment for green algae), novel nanocarriers (lipid nanoparticles mimicking mRNA vaccine delivery, cell-penetrating peptide conjugates), and optimized RNP delivery protocols to minimize toxicity and maximize editing efficiency without genomic integration [8,17]. Overcoming this “algal fortress” is paramount for translating CRISPR tools into practical metabolic engineering success. Delivery efficiency varies dramatically between species. Electroporation, while established for *C. reinhardtii*, often yields low transformation frequencies and high mortality in diatoms or *Nannochloropsis* due to their robust silica- or algaenan-based cell walls [17,36,37]. Biolistics can be species-agnostic but frequently leads to multi-copy insertions and silencing in *Nannochloropsis*, complicating the generation of stable strains [38,39]. PEG-mediated transformation shows promise for *Haematococcus pluvialis* but requires extensive optimization for each strain [40]. Engineered viruses, highly efficient for some *Chlorella* species, lack broad applicability [41,42]. Consequently, novel strategies like cell wall-weakening enzymes or tailored nanocarriers are essential foci for non-model species.

#### 2.1.3. Harnessing and Optimizing Repair Mechanisms

The outcome of Cas nuclease-induced DSBs is dictated by the cell’s endogenous repair machinery, presenting both challenges and opportunities for moving beyond simple knockouts [43,44]. Non-Homologous End Joining (NHEJ) predominates in most microalgae, often leading to small indels suitable for gene knockouts. However, its error-prone nature is detrimental for precise insertions required for sophisticated pathway engineering. Microhomology-Mediated End Joining (MMEJ), utilizing short flanking homologous sequences, offers a more predictable alternative for small deletions but is less characterized in algae [45]. Homology-Directed Repair (HDR), essential for precise knock-ins or replacements, is notoriously inefficient in microalgae, especially in non-dividing cells common in dense cultures—a significant limitation directly impacting efforts to enhance “robustness, productivity, or novel pathway integration” [46]. To overcome the notorious inefficiency of HDR in microalgae, which critically limits precise knock-ins or replacements essential for advanced engineering goals like enhancing robustness, productivity, or novel pathway integration, several key strategies are employed. Enhancing HDR involves temporal control by expressing Cas9 specifically during S/G2 cell cycle phases using cell cycle-regulated promoters (e.g., histone H4 promoter) when HDR is active, and suppressing the dominant Non-Homologous End Joining (NHEJ) pathway through co-delivery of chemical inhibitors (e.g., SCR7 targeting DNA ligase IV) or expression of dominant-negative forms of core NHEJ factors (e.g., Ku70/80). Donor design is also optimized, such as using single-stranded oligodeoxynucleotides (ssODNs) instead of dsDNA in some species, incorporating asymmetric homology arms, and protecting donor ends from degradation via modifications like phosphorothioates [47,48]. Alternatively, NHEJ-dependent strategies exploit prevalent NHEJ activity to integrate large constructs, either through double-cut HDR (creating two flanking DSBs) or direct ‘NHEJ-capture’, where linearized donor DNA with compatible ends is ligated into the Cas-induced DSB [49,50]. Understanding and manipulating these repair pathways is fundamental for transitioning from simple knockouts to sophisticated genome engineering needed for orchestrating complex genetic rewiring and dynamic multi-gene circuits.

### 2.2. Transcriptional Regulation: CRISPR Activation and Interference (CRISPRa/i)

Catalytically deactivated Cas proteins (dCas9/dCas12) retain DNA-binding specificity but lack cleavage activity, embodying the move “beyond cutting”. By fusing them to transcriptional effector domains, researchers gain programmable control over gene expression without altering the underlying DNA sequence—a reversible and tunable approach perfectly suited for “dynamic regulation across multi-gene circuits” and “pathway balancing”.

#### 2.2.1. Core Principles

dCas9 serves as a modular scaffold. Fusion to transcriptional activators like VP64 (a tetramer of herpes simplex virus VP16 domain), p65 (NF-κB subunit), or Rta (Epstein–Barr virus transactivator) creates CRISPR activation (CRISPRa) systems [51,52]. These recruit the cellular transcription machinery (e.g., RNA polymerase II, co-activators) to the target promoter, boosting gene expression. Conversely, fusion to repressors like the Krüppel-associated box (KRAB) domain (recruiting heterochromatin-forming complexes) or the mSin3 interaction domain (SID4x) (recruiting histone deacetylases-HDACs) creates CRISPR interference (CRISPRi) systems, blocking transcription initiation or elongation [53,54,55]. Multi-domain activators (e.g., VPR:VP64-p65-Rta; SAM: synergistic activation mediator incorporating MS2-p65-HSF1) significantly enhance activation strength [56]. CRISPRa/i offers several key advantages crucial for metabolic engineering: reversibility (removal of the dCas9-effector complex restores baseline expression), tunability (effector expression level, sgRNA design targeting specific promoter elements like proximal vs. distal regions), multiplexing capability, and avoidance of permanent genomic changes—essential for studying essential genes, fine-tuning metabolic pathways (“elevating compound yields”), and avoiding the “pitfalls of uncontrolled random integration and positional effects often seen with traditional overexpression vectors” [57,58].

#### 2.2.2. Strategies and Case Studies in Microalgae

Implementing CRISPRa/i in microalgae requires careful optimization, reflecting the need for species-specific tool adaptation. Key considerations include choosing potent effector domains functional in the algal nucleus (often requiring testing), selecting effective target sites within promoter regions (empirical testing is common due to limited algal promoter annotation), and efficiently expressing the often-large dCas9-effector fusion proteins (codon optimization, strong promoters) [59]. Pioneering work in *C. reinhardtii* demonstrated successful dCas9-VP64-mediated activation of reporter genes (e.g., *ARS2*) and endogenous genes like *HSP70A* [60,61]. CRISPRi using dCas9-KRAB has effectively silenced transgenes and endogenous genes (e.g., *MAA7* encoding tryptophan synthase, conferring 5-fluoroindole resistance) [62,63,64,65]. More recently, multiplexed CRISPRi has been used to simultaneously repress multiple competing pathways (e.g., glycogen synthesis, β-glucan synthesis) in *Nannochloropsis oceanica*, successfully redirecting carbon flux towards lipid accumulation—a direct application for “boosting productivity” and “elevating compound yields” (lipids for biofuels) [66,67,68]. CRISPRa holds immense promise for activating silent biosynthetic clusters (common in diatoms), enhancing the expression of rate-limiting enzymes in pathways for high-value nutraceuticals like carotenoids (e.g., β-carotene, astaxanthin) or polyunsaturated fatty acids (PUFAs) such as EPA/DHA, or boosting stress response genes (e.g., heat shock proteins, antioxidant enzymes) for improved “robustness against stressors”. Inducible promoters controlling dCas9-effector expression add another layer of temporal control, enabling dynamic pathway induction aligned with growth phases or environmental conditions [9,69,70,71]. However, implementing robust CRISPRa/i in non-model species faces hurdles beyond model organisms like *C. reinhardtii* [72]. Activation strength using common effector domains (e.g., VP64) is often modest in species like *P. tricornutum* compared to the model, potentially due to differences in nuclear import, chromatin accessibility, or co-factor availability [73,74]. Identifying effective target sites within poorly annotated promoter regions of non-model algae requires extensive empirical testing [27,75]. Furthermore, expressing large dCas9-effector fusions (e.g., dCas9-VPR) can be more challenging in species with less efficient expression systems or compact genomes [76,77,78].

#### 2.2.3. Advantages

The non-mutagenic, reversible, and tunable nature of CRISPRa/i makes it uniquely suited for “dynamic metabolic engineering”, “pathway balancing”, and functional genomics studies, where permanent knockouts could be lethal or undesirable, directly addressing the need for nuanced interventions beyond simple knockouts [57,79,80].

### 2.3. Epigenome Engineering

Beyond the genetic code, eukaryotic gene expression is profoundly regulated by epigenetic marks—chemical modifications to DNA (cytosine methylation) and histone proteins (acetylation, methylation, phosphorylation). CRISPR-epigenome engineering uses dCas9 fused to epigenetic modifier enzymes to rewrite these marks at specific genomic loci, offering long-term, potentially heritable control over gene expression states—a powerful layer of “multi-layered interventions” for stable trait enhancement [81,82].

#### 2.3.1. Core Principles of Epigenome Editing

dCas9 serves as a precise targeting platform to guide epigenetic modifiers to specific genomic locations. For targeted DNA methylation, dCas9 is fused to DNMT3A to induce de novo DNA methylation, resulting in gene silencing, or to the catalytic domain of TET1 to promote DNA demethylation and subsequent gene activation [83,84]. For histone modifications, dCas9 directs writers, erasers, and readers to alter chromatin marks: fusions like dCas9-p300 core (a histone acetyltransferase) add activating H3K27ac marks, while dCas9-LSD1 removes activating H3K4me1/2 marks to cause repression, and dCas9-EZH2 (a component of PRC2) adds repressive H3K27me3 marks [85]. Collectively, these dCas9-mediated epigenetic modifications—whether altering DNA methylation or histone marks—fundamentally change chromatin accessibility (shifting between euchromatin and heterochromatin states), thereby directly recruiting or blocking the transcription machinery to regulate gene expression.

#### 2.3.2. Potential and Applications in Microalgae

CRISPR-based epigenome editing offers powerful tools to directly address challenges in microalgal engineering, such as transgene silencing. By precisely erasing repressive marks (e.g., H3K27me3) or adding activating marks (e.g., H3K27ac) at transgene loci or endogenous silent biosynthetic clusters, it can potentially overcome positional effects to achieve stable, high-level expression of transgenes or silenced pathways—essential for industrial viability, including the production of novel compounds like isoprenoids or toxins in diatoms [81,86,87,88]. Conversely, this technology enables the permanent silencing of undesirable genes, such as those in competing pathways (e.g., starch synthesis during lipid production), repressors of valuable pathways, or destabilizing transposable elements, by establishing repressive chromatin marks like DNA methylation or H3K27me3 [89]. Furthermore, it serves as a vital research tool to dissect the role of epigenetic regulation in algal development, stress adaptation (e.g., to heat, high light, salinity), and metabolism (e.g., lipid accumulation under nitrogen stress) through the targeted manipulation of marks [81,82,90]. Ultimately, it holds the promise to engineer stable epigenetic memory, creating heritable states that enhance key performance traits like stress tolerance or lipid production across generations without altering the DNA sequence, providing a novel route to develop robust industrial strains. Initial proof-of-concept studies targeting model loci are paving the way for this approach in diatoms (e.g., *Phaeodactylum*) and green algae (e.g., *Chlamydomonas*).

### 2.4. Base Editing

Base editors (BEs) represent a paradigm shift “beyond cutting”, enabling precise, targeted single-nucleotide changes without requiring DSBs, donor DNA templates, or reliance on inefficient HDR pathways. This is revolutionary for introducing point mutations, correcting pathogenic SNPs, or fine-tuning enzyme function—directly addressing the HDR bottleneck and enabling “base-level corrections” [91,92].

#### 2.4.1. Core Mechanisms of Base Editing

BEs consist of a catalytically impaired Cas nickase (nCas9 or nCas12), capable of nicking only one DNA strand, fused via a linker to a nucleobase deaminase enzyme. They function within a narrow “editing window” (typically positions 4–8 or 4–7, counting the PAM as 21–23) near the PAM site [93]. Cytosine base editors (CBEs) fuse nCas9 to a cytidine deaminase (e.g., rAPOBEC1, AID, evoFERNY), converting C•G base pairs within the window to U•G intermediates; cellular repair then converts U•G to T•A, resulting in a C•G to T•A transition, with high-efficiency variants (e.g., BE4max, evoAPOBEC1-BE4max) minimizing unwanted “bystander” C edits [94,95]. Conversely, Adenine Base Editors (ABEs) fuse nCas9 to an evolved E. coli TadA adenine deaminase (e.g., TadA-7.10, TadA-8e), converting A•T base pairs to inosine•T (I•T) intermediates that are repaired to G•C, achieving A•T to G•C transitions, as seen in optimized variants like ABE8e [96]. Dual Base Editors combine both cytidine and adenosine deaminase activities within a single protein for broader editing potential across the window. Representing a significant evolution beyond canonical base editors, Prime Editors (PEs) utilize a Cas nickase (usually H840A SpCas9) fused to a reverse transcriptase (RT) and a specialized prime editing Guide RNA (pegRNA). The pegRNA contains both the target-binding spacer and a 3′ extension encoding the desired edit(s) plus a primer binding site (PBS) [97]. The nCas9 nicks the target strand, and the RT uses the pegRNA extension as a template to synthesize a new DNA flap incorporating the edit(s) directly at the nicked site, which is then incorporated by cellular repair. This mechanism allows PEs to achieve the broadest editing scope: all 12 possible base-to-base transitions and transversions, as well as small insertions and deletions (up to ~80 bp), without creating double-strand breaks (DSBs) [98,99].

#### 2.4.2. Applications in Microalgae

BEs are ideally suited for microalgal engineering, effectively circumventing the HDR bottleneck. They enable precise enzyme optimization through targeted point mutations in key metabolic enzymes to enhance catalytic activity, alter substrate specificity, or reduce feedback inhibition; examples include mutating acetyl-CoA carboxylase (*ACCase*) to boost lipid synthesis flux, engineering RuBisCO for improved carbon fixation or reduced photorespiration, and optimizing desaturases/elongases for tailored PUFA profiles like DHA yield in *Nannochloropsis* [100,101]. Furthermore, BEs facilitate the creation of clean knockouts by introducing premature stop codons (e.g., converting CAA/CAG/CGA to TAA/TAG/TGA using CBEs), enabling efficient and predictable gene silencing without indels to disrupt competing pathways [102]. Additionally, they allow for strain repair by correcting deleterious mutations in elite strains arising from random mutagenesis or spontaneous events [103]. Finally, BEs permit the precise tuning of regulatory elements, such as modifying promoter sequences (e.g., TATA box, transcription factor binding sites) or regulatory RNA elements to fine-tune gene expression levels. Successful applications of base editing in microalgae like *C. reinhardtii* and *Phaeodactylum tricornutum* include introducing herbicide resistance mutations (e.g., in *psbA* conferring resistance to atrazine or diuron) and creating precise tags [104,105].

#### 2.4.3. Advantages and Limitations

BEs offers significant advantages over traditional CRISPR-Cas9 editing, primarily because its DSB-free mechanism minimizes genotoxicity and chromosomal rearrangements [106]. It achieves high efficiencies in amenable loci without requiring donor DNA and enables the editing of non-dividing cells, resulting in cleaner, more predictable outcomes than NHEJ-mediated knockouts [104,106]. However, the technology faces limitations: its application is restricted by PAM availability and a narrow editing window. Within this window, unwanted bystander edits (C or A conversions for CBEs/ABEs) can occur, alongside potential off-target DNA editing (typically exhibiting off-target frequencies of 0.1–1.0% in standard systems), though this is reduced by 10- to 100-fold to <0.01% in newer high-fidelity variants [107]. Off-targeted RNA editing is mediated by the deaminase domain. Insertion capabilities are constrained by a limited payload size, especially with standard base editors compared to prime editors (PEs), and efficiency varies significantly depending on sequence context, chromatin state (with heterochromatin being refractory), and cell type [108,109]. While PEs offer greater versatility, they can be less efficient than CBEs/ABEs for simple transitions and require complex pegRNA design. Consequently, ongoing engineering efforts focus on expanding PAM compatibility (e.g., SpG, SpRY, Sc++ Cas9 variants recognizing NGN, NRN), narrowing editing windows and reducing bystander effects (e.g., SECURE-BEs), developing Cas12-based base editors, and improving PE efficiency and delivery [97,110].

### 2.5. Prime Editing: Expanding the Horizon of Precision

Prime editing (PE) represents a transformative leap beyond conventional base editing and HDR, enabling versatile DNA alterations without double-strand breaks (DSBs) or donor DNA templates, embodying the ultimate precision toolkit [111,112]. The PE system comprises an engineered Cas9 nickase (H840A)-reverse transcriptase (RT) fusion protein guided by a prime editing guide RNA (pegRNA). The pegRNA simultaneously specifies the target site via its spacer and encodes the desired edit via its 3′ extension, which contains the primer binding site (PBS) and the reverse transcriptase template (RTT) [113]. Upon binding, the nickase cleaves the non-complementary (target) DNA strand, and the RT synthesizes a new DNA flap using the RTT as a template. Cellular DNA repair machinery (primarily involving DNA flap displacement and ligation) then incorporates this edited flap, facilitating precise insertions, deletions, and all 12 possible base substitutions [114,115].

#### Overcoming Limitations in Microalgae

While base editors (CBEs/ABEs) are constrained by PAM availability, editing windows, and bystander edits, PE offers significantly broader targeting scope and edit diversity, crucial for complex metabolic engineering. Its DSB-free nature minimizes genotoxicity and chromosomal instability—critical advantages for microalgae where DSB repair pathways, especially HDR, are notoriously inefficient and error-prone [65,116,117,118]. PE circumvents the need for HDR entirely, making it ideal for introducing complex edits like multiple adjacent point mutations, codon replacements, small indels in regulatory regions, or repairing disease-associated mutations in key metabolic genes [119,120]. Initial success has been demonstrated in *Arabidopsis thaliana* cells, where PE achieved an average 1.15% editing efficiency, which is 16.4-fold higher than previously reported [121,122,123]. Challenges remain in pegRNA design optimization (algorithms like pegFinder and PrimeDesign are improving this), delivery efficiency for the large PE fusion protein (~6.3 kb for PE2), and achieving high efficiencies comparable to base editors in some contexts. However, its unique ability to perform “search-and-replace” editing positions PE as the gold standard for precision genome engineering in recalcitrant algal systems, enabling edits previously impossible or extremely difficult with other methods [124,125].

### 2.6. Multiplexed Genome Engineering: Rewiring Complex Pathways

Engineering the complex genetic rewiring required for next-generation microalgal metabolic engineering—such as elevating compound yields or engineering intricate pathways—necessitates simultaneous modification of multiple genomic loci [28,72]. This task is ideally addressed by multiplexed CRISPR systems, which co-deliver several guide RNAs (gRNAs) targeting distinct genes within a single cell. Key enabling strategies include polycistronic gRNA arrays, where flanked gRNAs are processed by endogenous RNases P and Z, or ribozyme-based systems that self-cleave from primary transcripts—both proven highly efficient in diatoms [28,62]. Alternatively, CRISPR array systems exploit the native multi-crRNA processing of Cas12a, where a single transcript with crRNAs separated by direct repeats is cleaved into individual units [126]. Orthogonal Cas protein co-expression employs distinct Cas proteins with cognate gRNAs from separate vectors/promoters to target different loci simultaneously, minimizing cross-talk and enabling independent regulation [127,128]. This multiplexing capability is indispensable for accelerating metabolic reconstruction: it allows for knocking out competing pathways, stacking beneficial traits (combining edits for growth, stress tolerance, and product accumulation in one transformation cycle), and dynamic pathway balancing via multiplexed CRISPRa/i to coordinate enzyme expression levels (e.g., fine-tuning phytoene synthase, lycopene cyclase, and beta-carotene hydroxylase genes to optimize astaxanthin flux) [129,130,131].

### 2.7. CRISPR Logic Gates and Biosensors: Enabling Smart Control

Integrating CRISPR components with biosensors creates sophisticated genetic circuits for autonomous, dynamic regulation of algal metabolism in response to environmental or metabolic cues—realizing the vision of “dynamic regulation across multi-gene circuits” for industrial robustness.

#### 2.7.1. CRISPR-Based Logic Gates

Engineered gRNAs or dCas regulators enable programmable genetic switches that introduce Boolean logic into cellular control [132,133]. This programmability is achieved through distinct mechanisms: gRNA switches incorporate regulatory elements like riboswitches or protease cleavage sites directly within the gRNA scaffold; the presence of the specific small molecule or protease then controls gRNA activity by inducing folding or cleavage, rendering it active or inactive, respectively [134,135,136]. Beyond simple switches, dCas-based systems can implement more complex logic, such as AND gates. These require the co-localization of split activator fragments at a single target promoter, where each fragment is fused to a different dCas protein (e.g., dCas9 and dCas12a) guided by distinct gRNAs [136]. Crucially, the functional activator complex only forms, and thus expression is only driven, when both gRNAs are present and bind adjacent sites, ensuring activation occurs solely under specific combinatorial conditions [137].

#### 2.7.2. Biosensor-Integrated Control for Closed-Loop Regulation

Coupling endogenous algal sensors or synthetic sensors to CRISPR components enables context-dependent metabolic control, creating a closed-loop system that optimizes resource allocation and enhances robustness [138]. Nutrient-responsive control, achieved by linking starvation-inducible promoters to CRISPR effectors like dCas9-VP64 or CRISPRi, allows for targeted metabolic shifts [61,139]; for instance, activating lipid synthesis genes under N-limitation in *Nannochloropsis oceanica* increased lipid titers by 25% compared to constitutive overexpression [140,141]. This activation strategy also improved carbon partitioning efficiency to lipids [142,143]. Simultaneously, metabolite-responsive control utilizes synthetic biosensors to the trigger CRISPRi repression of competing pathways when precursor levels are high, e.g., malonyl-CoA biosensor-mediated repression in *Chlamydomonas reinhardtii*, elevating triacylglycerol (TAG) accumulation by 30–50% without compromising biomass yield [144,145,146,147,148,149]. Further precision is added through optogenetic control, where fusing CRISPR activators/repressors to algal or fungal photoreceptors enables light-inducible regulation [146]; specific wavelengths or pulses can activate pathways like carotenoid biosynthesis (red light), boasting 2.1-fold higher β-carotene yields [150,151,152], or repress others like starch synthesis (blue light) with high temporal resolution, aligning production with diurnal cycles or process demands [153]. This integrated approach minimizes metabolic burden, prevents intermediate toxicity, and enhances product titers under fluctuating industrial conditions, directly contributing to economic viability.

### 2.8. CRISPR-Enabled Directed Evolution: Accelerating Strain Optimization

CRISPR tools dramatically accelerate the traditionally “slow and often unpredictable” pace of algal strain improvement by enabling high-throughput functional genomics and directed evolution. Genome-wide or pathway-specific sgRNA libraries facilitate pooled CRISPR interference (CRISPRi) and activation (CRISPRa) screens in algal populations transformed with dCas9-effector fusions [154]. CRISPRi knockdown libraries identify essential genes through growth defects or uncover negative regulators of desirable traits, such as repression-enhancing lipid overproduction or stress tolerance, as demonstrated in *C. reinhardtii* screens, revealing novel regulators of photosynthesis and lipid metabolism [101,155]. Conversely, CRISPRa activation libraries uncover genes whose overexpression boosts performance, like stress tolerance or pigment yield, by activating cryptic biosynthetic genes or transporters to reveal novel pathways or bottlenecks [9]. Beyond screening, CRISPR is leveraged for targeted mutagenesis and directed evolution. Techniques include CRISPR-Cas9 Assisted Recombineering (CRASAR), which induces targeted double-strand breaks near a gene concurrent with random mutagenesis, exploiting cellular repair to enrich mutations at the specific locus [156]. Alternatively, dCas9 can be fused to hyperactive error-prone DNA polymerases or cytidine deaminases to create localized hypermutation zones at target genes, generating diverse allele libraries under selection for phenotypes like improved enzyme kinetics [101]. Furthermore, CRISPRa-driven evolution uses dCas9-VPR to overexpress mutagenic enzymes while simultaneously activating a target pathway, diversifying key enzymes within the pathway under selection pressure to rapidly evolve optimized variants [157].

These approaches enable a rapid evolution of algal strains with optimized enzyme kinetics, enhanced pathway flux, or novel functions, bypassing the slow pace of natural evolution and the low efficiency of traditional random mutagenesis. Integrating multiplexed editing, biosensors, and directed evolution creates a powerful, integrated pipeline for developing next-generation algal cell factories with the “industrial robustness” and performance required for economic viability [57,158,159].

The CRISPR toolkit for microalgae has rapidly evolved from a simple DNA cleaver into the sophisticated synthetic biology arsenal envisioned in the Introduction. The foundational precision of engineered Cas variants, coupled with relentless innovations in delivery and repair pathway manipulation, underpins the revolutionary advanced applications now possible. CRISPRa/i provides a powerful lever for reversible, tunable transcriptional control, ideal for dynamic pathway balancing and optimizing flux towards high-value compounds. Epigenome editing opens the door to potentially stable, long-term reprogramming of gene expression states by rewriting the chromatin landscape, offering solutions to transgene silencing and enabling stable trait enhancement. Base editors, particularly CBEs and ABEs, offer a remarkably efficient route for introducing precise point mutations to optimize enzymes or create clean knockouts, effectively bypassing the notorious HDR bottleneck in algae. Prime editors promise even greater versatility for diverse edits, representing the pinnacle of DSB-free precision. Multiplexing strategies are essential for rewiring complex metabolic networks by simultaneously targeting multiple genes, while CRISPR logic gates and biosensors enable smart, autonomous control of metabolism in response to environmental and metabolic cues. Finally, CRISPR-enabled directed evolution dramatically accelerates the optimization of enzymes and pathways.

The strategic integration of these tools—using CRISPRi to silence competing pathways, base editing to optimize key enzyme kinetics, CRISPRa to boost flux through engineered routes, multiplexing to coordinate these changes, epigenome editing for stable enhancement, and biosensors for dynamic control—exemplifies the “Swiss Army Knife” approach [101,160]. This multi-layered, programmable control is essential for tackling the complex, interconnected challenges of next-generation microalgal metabolic engineering: achieving industrial-scale productivity, resilience against biotic and abiotic stresses, and high titers of diverse valuable compounds as demanded for true economic viability (Figure 1). While challenges in delivery efficiency, species-specific optimization, off-target effects, and scalability remain active frontiers of research, the core CRISPR toolkit beyond cutting has irrevocably transformed the prospects for microalgae as sustainable, high-performance cellular factories. The ongoing refinement and creative deployment of these tools will be central to unlocking their full potential for driving a circular bioeconomy (Table 1).

## 3. Future-Oriented Applications: Integrated CRISPR Strategies for Next-Generation Metabolic Engineering

The foundational CRISPR toolkit described above provides unprecedented precision for addressing core challenges in microalgal biotechnology. Looking forward, this section synthesizes prospective applications where integrated, multi-tool strategies could transform metabolic engineering objectives: enhancing photosynthesis, optimizing high-value compound production, and improving stress resilience in next-generation industrial settings. Rather than reporting established implementations, we outline future pathways for leveraging CRISPR’s synergistic potential to overcome persistent bottlenecks at the commercial scale.

### 3.1. Future Photosynthesis Enhancement: Multi-Layered CRISPR Strategies

CRISPR technologies provide powerful, multi-layered strategies to enhance photosynthetic efficiency and biomass accumulation, forming the foundation for economically viable industrial cultivation [189,190,191]. For photosynthesis optimization, CRISPRi enables the precise tuning of targeted gene expression, boosting photosystem repair components under stress [30]. Base editing optimizes Carbon Concentrating Mechanisms (CCMs) by introducing point mutations into components like carbonic anhydrases and bicarbonate transporters to enhance CO_2_ affinity under varying industrial conditions, with multiplexed CRISPRi repressing competing carbon sinks [192]. Further, CRISPRi knockdown of photorespiratory enzymes minimizes carbon loss, CRISPRa enhances engineered photorespiration bypass pathways, and base/prime editing fine-tunes key carbon flux enzymes (e.g., *PEPC*) to favor biomass accumulation [101]. Base/prime editing can also be used to enhance photoprotection by introducing mutations into NPQ-related proteins to alter kinetics, while CRISPRa boosts genes for rapid NPQ recovery [193]. Beyond photosynthesis, CRISPR enables multi-layered control over lipid metabolism for optimized biofuel production. Multiplexed CRISPRi represses competing pathways, CRISPRa boosts key lipid synthesis enzymes, and base editing optimizes enzyme kinetics or knocks out lipases degrading TAG [194]. Base editing tailors’ fatty acid profiles by modifying thioesterases and desaturases, with CRISPRa/i fine-tuning their relative expression levels. Finally, CRISPR biosensors and optogenetic systems enable dynamic, closed-loop control of lipid induction, such as activating TAG synthesis only under nitrogen starvation or using specific light wavelengths for precise timing within photobioreactors [158,159].

### 3.2. Next-Generation Lipid/Biofuel Optimization: Integrated Control Systems

CRISPR enables the multi-layered optimization of lipid and biofuel production through precise control over the entire metabolic network [195]. Multiplexed CRISPR strategies are essential for this, allowing for the simultaneous repression of competing pathways like starch or glycogen synthesis via CRISPRi, while CRISPRa boosts key enzymes involved in fatty acid synthesis, triacylglycerol (TAG) assembly, and lipid droplet stabilization [196]. Base editing could further optimize enzyme kinetics, such as reducing feedback inhibition in *ACCase*, or create clean knockouts of lipases to prevent TAG degradation. Furthermore, CRISPR facilitates tailoring fatty acid profiles: base editing modifies the substrate specificity and activity of thioesterases (*TEs*) and desaturases to control chain length and unsaturation, while CRISPRa/i fine-tunes the relative expression levels of TEs, desaturases, and elongases to achieve desired profiles like high oleic acid or specific PUFAs. Critically, CRISPR biosensor-logic gates enable dynamic, closed-loop induction under stress conditions. For instance, an N-starvation-inducible promoter driving dCas9-VP64 activates TAG synthesis and represses lipid catabolism genes specifically under nitrogen limitation. Similarly, a malonyl-CoA biosensor activates elongases and represses competing pathways when precursor levels peak. Optogenetic CRISPR systems, using dCas9-effector-photoreceptor fusions and specific light wavelengths, provide precise temporal control over lipid induction within photobioreactors.

### 3.3. Advanced Biosynthesis: Engineering High-Value Compound Pathways

CRISPR tools enable the overproduction of diverse high-value natural compounds and the engineering of novel biosynthetic pathways [101]. For pigments like carotenoids and phycobiliproteins, multiplexed CRISPRa coordinately upregulates pathway enzymes, while CRISPRi represses competing pathways. Base editing could be used to remove feedback inhibition sites or optimize enzyme kinetics, and epigenetic editing stabilizes the expression of heterologous bilin pathways. In polyunsaturated fatty acid (PUFA) biosynthesis, base editing can enhance the activity, specificity, and stability of elongases (*ELOVL*) and desaturases (*FAD*), CRISPRa boosts rate-limiting enzymes (e.g., *Δ6-desaturase*), and multiplexed CRISPRi represses competing fatty acid utilization pathways (e.g., β-oxidation) [197]. For valuable proteins and peptides, CRISPRa significantly enhances endogenous protein expression, while prime editing (PE) enables the scarless integration of large heterologous expression cassettes into genomic safe harbors, complemented by epigenome editing to maintain an open chromatin state for stable, high-level expression [6,9]. Similarly, in terpenoid, vitamin, and antioxidant production, CRISPRi knocks down competing pathways, CRISPRa activates rate-limiting steps, and base editing optimizes key enzymes [9]. Engineering novel pathways further leverages multiplexed PE for precise heterologous gene integration and CRISPRa for coordinated expression (Figure 1).

### 3.4. Prospective Stress Resilience Engineering

CRISPR technologies enhance microbial stress tolerance and robustness for industrial cultivation through multiple approaches [28,90]. For specific stress resistance, CRISPR activation boosts protective gene expression—such as antioxidant enzymes against oxidative stress, heat shock proteins for heat stress, and ion transporters/compatible solute synthases for salt stress—while base editing generates hyperactive alleles or removes regulatory constraints [198,199,200]. Additionally, cell wall and membrane composition can be optimized using CRISPR interference (CRISPRi) or base editing to knockout rigid cell wall synthesis genes, facilitating downstream extraction, or through CRISPRa/base editing to enhance desaturase expression for increased membrane unsaturation and cold tolerance; engineering novel polymers may further improve shear resistance [201]. Beyond targeted edits, CRISPR-enabled screening accelerates tolerance engineering: genome-wide CRISPRi/CRISPRa knockout/activation libraries enable functional genomics under stress, identifying negative regulators to knockdown or protective genes to overexpress. Furthermore, targeted dCas9-mutagenesis tools expedite the directed evolution of stress-resistant variants [202].

### 3.5. Future Carbon Utilization Strategies

Optimizing carbon capture and utilization requires maximizing carbon fixation and directing metabolic flux for enhanced sustainability and productivity. Enhancing CO_2_ fixation efficiency is achieved through techniques like base editing to introduce beneficial mutations into *Rubisco*, CRISPRa to boost *Rubisco activase* or *CCM* components, and epigenome editing to potentially unlock higher endogenous *Rubisco* expression [15,203,204]. Furthermore, directing carbon flux involves multiplexed CRISPRi to repress major competing carbon sinks and CRISPRa to enhance flux into desired target pathways; CRISPR-based logic gates offer dynamic control, rerouting carbon based on cellular status [101]. Additionally, broadening the utilization of alternative carbon sources is engineered using base or prime editing to broaden the substrate specificity of transporters or catabolic enzymes, employing CRISPRa to boost the expression of endogenous transporters and enzymes, and utilizing prime editing for the precise integration and expression of heterologous pathways, such as those enabling fungal *xylose* utilization [205,206,207] (Figure 1).

### 3.6. Forward-Looking Industrial Strain Design

The integrated CRISPR toolkit enables the comprehensive engineering of industrial algal strains for lipid biofuel production by strategically combining diverse functionalities. CRISPRi, implemented through multiplexed tRNA-gRNA arrays, can be used to simultaneously silence competing pathways including starch synthesis genes, β-glucan synthesis, and key lipases [66,208,209]. Base editing can be used to optimize the expression of critical enzymes like *ACCase*, reducing feedback inhibition via specific point mutations, and improves the kinetics/profile of a key desaturase [196,210,211]. CRISPRa is dynamically controlled, utilizing an N-starvation-inducible promoter; this activates lipid assembly genes and represses β-oxidation genes specifically under nitrogen limitation, with precursor availability further fine-tuned by a malonyl-CoA biosensor [212]. Epigenome editing targets the promoters of engineered lipid synthesis genes to maintain an open chromatin state, ensuring stable, high-level expression across generations and preventing silencing [6,213]. Prime editing (PE) precisely integrates a construct for a high-efficiency Rubisco variant, including its promoter, ORF, and terminator, into a genomic safe harbor locus to avoid positional effects [6,214]. Finally, CRISPRa boosts the expression of a small heat shock protein to confer enhanced thermotolerance crucial for outdoor cultivation [215].

This prospective multi-layered intervention exemplifies future workflows moving beyond simple gene knockouts. When fully realized, CRISPR-driven synthetic biology unlocks the potential to create microalgal cell factories with the unprecedented productivity, resilience, and product specificity demanded for a sustainable circular bioeconomy (Figure 1). While challenges in delivery efficiency, species-specific tool optimization, off-target effects, and scalability remain active frontiers, the integrated toolkit outlined here provides a clear and powerful roadmap for realizing the full potential of next-generation microalgal metabolic engineering (Table 2).

## 4. Challenges, Limitations, and Future Perspectives

The transformative potential of CRISPR-driven synthetic biology for microalgal metabolic engineering, moving decisively beyond simple DNA cutting, is undeniable. As detailed in previous sections, tools like CRISPRa/i, base/prime editing, epigenome engineering, multiplexed systems, and biosensor-integrated logic gates offer unprecedented capabilities to engineer industrial-grade strains with enhanced productivity, resilience, and tailored product profiles. However, significant hurdles remain to fully unlock this potential and translate laboratory successes into robust, economically viable biomanufacturing platforms. This section critically examines the persistent challenges, outlines strategies for toolkit refinement, and explores future directions essential for realizing the vision of microalgae as cornerstones of a sustainable circular bioeconomy.

### 4.1. Microalgae-Specific Challenges

The inherent biological diversity and complexity of microalgae present unique obstacles that demand species-specific solutions, directly impacting the deployment of the CRISPR toolkit.

#### 4.1.1. Genetic Diversity and Tool Compatibility

Microalgae encompass vast phylogenetic diversity (green algae, diatoms, eustigmatophytes, red algae, etc.), leading to profound differences in genome architecture, chromatin organization, DNA repair machinery, and cellular physiology. As highlighted before, the efficiency and specificity of CRISPR components (Cas variants, gRNAs) vary dramatically between species [240,241]. For instance, while *Chlamydomonas reinhardtii* has become a model, industrially relevant strains like *Nannochloropsis* spp., *Phaeodactylum tricornutum*, *Haematococcus pluvialis*, or *Chlorella* spp. exhibit distinct Cas protein preferences (e.g., Cas12a often outperforms Cas9 in diatoms), PAM requirements, and tolerance to foreign DNA/RNP delivery [28]. Endogenous repair pathways, particularly the notoriously inefficient Homology-Directed Repair (HDR) crucial for precise knock-ins and a major bottleneck, differ significantly [9,242]. This necessitates laborious empirical optimization of Cas orthologs, codon usage, promoters (viral, endogenous strong/inducible), and repair pathway manipulation strategies for each target species, hindering rapid tool transfer [243].

Concrete differences in CRISPR tool performance emerge across microalgae species. While SpCas9 remains standard in *C. reinhardtii*, Cas12a variants consistently achieve higher editing efficiency in diatoms (*P. tricornutum*) and eustigmatophytes. HDR efficiency is generally very low (<1%) in most microalgae but varies by species; rates in *C. reinhardtii* can be boosted to ~5–10% using NHEJ inhibition and cell cycle synchronization, whereas *N. gaditana* or *P. tricornutum* often persist < 1%, necessitating alternative strategies like NHEJ-mediated integration or prime editing [7,244]. For transcriptional activation, dCas9-VP64 functions well in *C. reinhardtii*, but achieving strong, consistent activation in *H. pluvialis* or *N. oceanica* is more challenging, typically requiring more potent multi-domain activators like VPR or SAM; however, their effective application requires species-specific validation [45,181]. Furthermore, the burden of tool optimization is evident in promoter choice: viral promoters (e.g., SV40, CMV) work in some *Chlamydomonas* strains but often fail in diatoms or *Nannochloropsis*, demanding extensive screening of endogenous promoters such as *PtUbiquitin* in *P. tricornutum* or *NoActin* in *N. oceanica* (Table 3) [37,245,246]. Table 3 highlights the significant variability in baseline tool performance and requirements. Overcoming these species-specific hurdles requires dedicated optimization efforts for each chassis, moving beyond protocols established solely in *C. reinhardtii*.

#### 4.1.2. The Formidable Cell Wall Barrier

Efficient intracellular delivery of CRISPR components remains arguably the single most significant bottleneck. Microalgae possess diverse, often recalcitrant, cell wall compositions and polysaccharide capsules, forming a robust “algal fortress” [17,265]. Physical methods are species-agnostic but suffer from low efficiency, high cell death, and multi-copy integration causing silencing. Chemical methods and biological vectors require extensive customization. While innovations like cell wall-weakening enzymes, advanced nanocarriers, and optimized RNP protocols show promise, a universally efficient, low-toxicity, high-throughput delivery method suitable for diverse industrially relevant algae is still lacking.

#### 4.1.3. Transformation and Screening Inefficiency

Low transformation frequencies, even in model species, coupled with the lack of efficient, scalable screening methods, severely limit throughput. Reliance on antibiotic/herbicide resistance markers is unsustainable for multiplexed engineering and raises regulatory concerns [28,29]. Developing robust, versatile selection markers or, ideally, efficient marker-free strategies is crucial. High-throughput phenotyping platforms for traits like lipid content, pigment yield, or stress resilience are also needed to match the potential of multiplexed CRISPR libraries [160,266].

#### 4.1.4. Endogenous CRISPR System Interference

Some microalgae possess native CRISPR-Cas systems. The interaction between endogenous systems and introduced CRISPR tools is poorly understood but could potentially lead to self-targeting, reduced efficiency, or unexpected immune responses. Characterizing native systems and designing exogenous tools to avoid cross-reactivity is an emerging consideration [8,210].

#### 4.1.5. Inadequate Chassis Knowledge

Despite progress, the genomic, transcriptomic, proteomic, and metabolic annotation of many non-model, industrially promising microalgae remains incomplete. Gaps in understanding essential genes, regulatory networks, metabolic flux distributions, and stress response pathways impede rational target selection for CRISPR interventions. Genome-scale metabolic modeling (GMM) offers a powerful computational framework to accelerate chassis characterization. By integrating multi-omics data with constraint-based reconstruction (e.g., GSM for *Nannochloropsis*), GMM can predict the following [267,268]: (1) essential/redundant genes for knockout prioritization [269], (2) metabolic flux bottlenecks for CRISPRa/i targeting [79,268], and (3) systemic impacts of pathway engineering prior to experimental validation [270,271]. The comprehensive multi-omics characterization of key algal chassis is enhanced by GMM-guided hypothesis generation and remains fundamental for effective CRISPR-based design [28,272,273].

### 4.2. Toolkit Refinement

While the core CRISPR toolbox is established, continuous innovation is needed to enhance its precision, power, versatility, and applicability for the complex demands of algal metabolic engineering.

#### 4.2.1. Enhancing Efficiency, Precision, and Specificity

Advancing CRISPR technologies in algae necessitates multifaceted progress across core components. First, engineering novel Cas variants is vital for broader targeting and easier delivery, particularly in species with compact genomes or rigid cell walls; this includes developing smaller proteins with flexible PAM requirements and systematically evaluating high-fidelity variants to minimize off-target effects across diverse algae [6,28]. Additionally, gRNA design optimization requires improved computational algorithms trained on algae-specific data to accurately predict on-target efficiency and reduce off-target activity, incorporating critical factors like species-specific chromatin accessibility and DNA sequence context [9]. Ultimately, mastering endogenous repair pathways—including NHEJ, MMEJ, and HDR—remains critical. This involves developing more effective and less toxic NHEJ inhibitors, refining cell cycle synchronization strategies to favor HDR, optimizing donor DNA designs for enhanced knock-in efficiency, and exploring transformative approaches like alternative repair pathways or synthetic repair machineries [274].

#### 4.2.2. Expanding the Editing Repertoire

Significant advancements are essential across multiple fronts to fully realize CRISPR’s potential for algal engineering. Enhancing CRISPR activation (CRISPRa) and interference (CRISPRi) systems is critical, as current algal CRISPRa often suffers from modest activation levels—typically achieving only 2–5-fold induction in model systems like *Chlamydomonas reinhardtii* and rarely exceeding 10-fold even with multi-effector arrays (VPR/SAM) in industrially relevant *Nannochloropsis* spp., substantially lower than the 50–100-fold common in mammalian or bacterial systems [6,124,275,276]. This necessitates developing more potent synthetic effector domains, optimized multi-effector arrays beyond existing systems like VPR/SAM, robust inducible systems with tighter control and wider dynamic range, and similarly more potent and specific CRISPRi repressors [277]. Optimizing base and prime editing is equally vital, involving the adaptation of next-generation base editors with narrowed editing windows, reduced bystander edits, expanded PAM compatibility (e.g., Cas12-based BEs), and improved heterochromatin efficiency. For prime editing (PE), key challenges include boosting its typically lower efficiency relative to base editors, optimizing pegRNA design, enhancing reverse transcriptase processivity, developing efficient delivery strategies for the large PE fusion protein (~6.3 kb), and demonstrating robust PE for larger insertions/deletions and complex edits in diverse algae [46,278]. Exploring new CRISPR-derived tools, such as CRISPR-based RNA editing, CRISPR-associated transposases (CAST) for targeted large insertions, or CRISPR-guided recombinases, holds future promise for alternative pathway integration and regulation strategies. Improving tunability and orthogonality is fundamental for engineering complex metabolic pathways, demanding precise, dynamic, and independent control over multiple genes; this necessitates enhancing orthogonality and developing more sophisticated tunable systems to achieve finer spatiotemporal resolution and minimize cross-talk in multiplexed circuits [197,272]. Finally, mitigating off-target effects remains an ongoing priority, requiring comprehensive off-target assessment in algae using adapted methods like CIRCLE-seq or GUIDE-seq, integrating findings into gRNA design algorithms, employing strategies to suppress residual activity, and monitoring potential RNA off-target effects from deaminase domains in base editors [279] (Figure 1).

### 4.3. System Integration and Automation

Bridging CRISPR engineering with advanced computational and robotic platforms is essential for accelerating strain development. Deep integration of CRISPR tools with multi-omics enables rational design by identifying key regulatory nodes, rate-limiting steps, and novel gene functions through CRISPRi/a screening [280]. This omics-guided approach optimizes target selection and predicts the systemic impacts of interventions. Machine learning (ML) and artificial intelligence (AI) further revolutionize the process by predicting optimal gRNAs, pegRNAs, and CRISPRa/i targets; designing and optimizing de novo pathways and enzyme expression; modeling Cas variant performance; and linking genotypes to complex phenotypes [281,282]. To scale these capabilities, automated high-throughput platforms are indispensable: robotic systems automate DNA/RNP assembly, delivery, and transformation, while integrated screening platforms enable rapid, label-free phenotyping of thousands of clones [283,284]. Together, this synergy of multi-omics, ML/AI, and automation empowers CRISPR-enabled directed evolution (CRASAR, dCas9-mutagenesis) and functional genomics at scale, streamlining development from design to deployment.

### 4.4. Scaling up Applications and Industrial Considerations

Achieving marker-free, scarless editing (prime editing is particularly promising) is essential for regulatory simplicity and enabling sequential engineering. Navigating regulatory frameworks requires distinguishing between gene-edited and transgenic strains, conducting transparent science-based risk assessments, and proactive engagement with regulators to establish clear guidelines [285]. Finally, demonstrating economic viability through rigorous techno-economic analysis (TEA) to justify development costs via productivity gains, alongside comprehensive life cycle assessments (LCAs) validating environmental benefits, is crucial for commercialization and solidifying sustainability credentials [286,287].

### 4.5. Ethical and Biosafety Considerations

The responsible application of CRISPR technology, especially concerning environmental release, necessitates addressing significant concerns through rigorous Environmental Risk Assessment (ERA) and robust mitigation strategies [288]. A primary ERA focus is the potential impact of engineered algae escape cultivation, particularly the risk of engineered traits spreading to wild populations or other organisms via Horizontal Gene Transfer (HGT); although microalgae HGT rates are generally low, this requires rigorous assessment due to potential ecosystem disruption [289]. Furthermore, escaped algae could outcompete native species, alter nutrient cycles, or produce harmful compounds, making understanding the fitness cost of engineered traits in natural environments essential. Mitigating these escape risks is paramount and relies on multi-layered biocontainment: robust physical containment [290]; biological containment strategies such as engineering auxotrophies (dependence on supplied nutrients absent in nature) and inducible lethal genes (“kill switches” activated by environmental cues like the absence of an inducer) [290,291]; genetic use restriction technologies (GURTs) to prevent reproduction/persistence outside controlled conditions, noting that CRISPR itself can engineer sophisticated containment circuits [292]; and genetic isolation by minimizing pathogen-derived genes or antibiotic resistance markers while utilizing species with limited survival/hybridization potential locally [290,293,294]. Beyond biocontainment, commercializing CRISPR-engineered microalgae faces complex, fragmented regulatory landscapes that vary significantly by application and jurisdiction. For food and feed applications, products from edited strains often face classification as “Novel Foods” or “Bioengineered Foods”, requiring extensive safety dossiers including compositional, toxicological, and allergenicity assessments; engineered metabolic pathways invite additional scrutiny for unintended compounds. Furthermore, regions like the EU mandate GMO labeling regardless of detectability [295]. Environmental applications, such as open-pond cultivation, fall under stringent release frameworks, demanding exhaustive environmental risk assessments of invasiveness and gene flow [295,296]. Regulatory classification uncertainty persists: for example, the EU may classify transgene-free edited algae as GMOs, while the US employs a product-based approach [21,297]. Cross-jurisdictional challenges include divergent definitions of “genome-edited organism” across major markets, complicating global development. Additionally, technical difficulties in detecting small edits within complex algal matrices hinder traceability enforcement [298,299]. Navigating these hurdles requires proactive regulatory engagement during R&D, incorporating “biosafety by design” principles, and establishing open-access safety databases to reduce redundant testing [300,301]. Finally, Responsible Research and Innovation (RRI) underpins this effort, requiring proactive stakeholder engagement (public, policymakers, industry) in goals, benefits, and risks, coupled with research transparency, strict biosafety protocol adherence, and the development of ethical guidelines specific to algal biotechnology to foster public trust and ensure societal benefit. Continuous monitoring and adaptive management are crucial as the field advances (Figure 1).

### 4.6. Towards a CRISPR-Engineered Algal Future

The journey “beyond cutting” has equipped metabolic engineers with a remarkably versatile CRISPR synthetic biology toolkit, capable of precise genome rewriting, dynamic transcriptional control, epigenetic reprogramming, and sophisticated circuit engineering in microalgae. While significant microalgae-specific challenges—particularly genetic diversity, cell wall barriers, delivery efficiency, and incomplete chassis knowledge—hinder universal application, relentless innovation in Cas engineering, delivery methods, repair manipulation, and tool optimization is rapidly narrowing these gaps. The future lies in the intelligent integration of these refined tools with multi-omics insights, ML/AI-driven design, and automated high-throughput platforms, enabling the rational construction of algal cell factories with unprecedented capabilities. Expanding applications to diverse, industrially relevant species, ensuring genetic stability and scalability, navigating evolving regulations, and rigorously addressing economic viability and environmental biosafety through ERA and biocontainment are critical steps for translation. By embracing Responsible Research and Innovation, the field can harness the full power of CRISPR-driven synthetic biology to propel microalgae beyond their current limitations, realizing their immense potential as sustainable, programmable, and economically viable platforms for next-generation biomanufacturing within a circular bioeconomy (Figure 1). The path forward demands continued interdisciplinary collaboration, focusing on overcoming the outlined challenges to unlock the true industrial potential of these photosynthetic powerhouses.

## 5. Conclusions

CRISPR technology has evolved into a multifaceted synthetic biology toolkit that transcends its original role as a programmable DNA-cutting instrument. This transformation empowers metabolic engineers to overcome longstanding barriers in microalgal engineering through key advancements. Precision transcriptional control (CRISPRa/i) enables tunable gene activation or repression for dynamic pathway optimization, exemplified by boosted lipid yields via carbon sink repression. Beyond transcriptional regulation, epigenome engineering rewrites epigenetic marks to combat transgene silencing and stabilize trait expression in industrial strains. DSB-free precision editing tools—including base and prime editors—achieve targeted nucleotide changes, insertions, or deletions without double-strand breaks, facilitating enzyme optimization and herbicide resistance. Multiplexed genome engineering further extends its capabilities, allowing the simultaneous rewiring of metabolic networks through knockout of competing pathways and trait stacking. CRISPR biosensors and logic gates introduce autonomous, dynamic control of metabolism in response to environmental cues (e.g., nutrient-responsive lipid synthesis), while CRISPR-enabled directed evolution accelerates strain optimization via high-throughput screening and targeted mutagenesis.

The integrated CRISPR toolkit—transcending its origins as molecular “scissors”—now provides the precision, programmability, and versatility essential for engineering microalgae into next-generation biomanufacturing platforms. By enabling the predictable redesign of photosynthesis, carbon flux, stress resilience, and product specificity, CRISPR-driven synthetic biology unlocks the path toward economically viable, sustainable algal cell factories capable of supporting a circular bioeconomy. Realizing this potential demands continued interdisciplinary collaboration to overcome existing barriers, refine tool efficacy, and demonstrate scalable industrial applications.

## Figures and Tables

**Table 1:** CRISPR toolkit beyond cutting: core components for microalgal metabolic engineering.

Tool Category	Core Mechanism	Key Applications	Key Advantages	Main Limitations	Ref.
CRISPRa/i	dCas9 fused to activators/repressors	- Dynamic pathway balancing - Essential gene modulation	- Reversible/tunable - No DNA changes	- Modest activation strength - Large fusion delivery	[69,133,161,162]
Epigenome Editing	dCas9 fused to epigenetic modifiers	- Stable trait enhancement - Transgene desilencing	- Heritable reprogramming - Overcomes position effects	- Limited algal characterization - Chromatin-dependent efficiency	[27,87,89,163,164,165]
Base Editors	nCas9 fused to deaminase	- Enzyme optimization - Clean knockouts	- DSB-free - High efficiency - Bypasses HDR	- PAM/window restrictions - Bystander edits	[166,167,168,169,170,171,172]
Prime Editors	nCas9-RT + pegRNA	- Diverse edits (indels, substitutions)	- Broad editing scope - No donor DNA	- Low efficiency - Complex pegRNA design	[6,45,170,171,172,173,174]
Multiplexed Systems	tRNA-gRNA arrays; Orthogonal Cas	- Coordinated pathway rewiring - Trait stacking	- Simultaneous multi-gene editing	- Cross-talk risk - Species-specific optimization	[90,175,176,177,178,179,180,181]
CRISPR Biosensors/Logic Gates	gRNA switches; dCas-split effectors	- Nutrient-responsive induction - Precise temporal control	- Autonomous regulation - Minimizes metabolic burden	- Few algal biosensors - Circuit tuning required	[69,159,182,183,184,185,186,187,188]

**Table 2:** Potential applications of the CRISPR toolkit in next-generation microalgal metabolic engineering.

Engineering Goal	Potential CRISPR Tool(s) Applied	Strategy	Key Outcomes	Ref.
Enhanced Photosynthesis and Biomass	CRISPRi + Base editing + Multiplexing	- Tune LHC genes (reduce antenna size). - Optimize CCMs via point mutations. - Repress photorespiration.	Improved photon penetration; 10% ↑ CO_2_ fixation; reduced carbon loss.	[28,101,156,216,217,218]
Lipid/Biofuel Optimization	Multiplexed CRISPRi + CRISPRa + Base editing + Biosensors	- Silence starch synthesis. - Engineer thioesterases for tailored FAs. - N-starvation-inducible lipid induction.	>25% lipid yield ↑ in *Nannochloropsis*; dynamic flux control; high-oleic acid profiles.	[160,204,219,220,221,222,223]
High-Value Compound Synthesis	CRISPRa + Base editing + Epigenome editing + PE	- Activate carotenoid pathway (PSY, LCYB); repress chlorophyll. - Optimize desaturases (PUFAs). - Integrate heterologous pathways (PE).	Stable astaxanthin flux; enhanced EPA/DHA; scarless protein expression.	[6,9,101,188,197,224,225,226]
Stress Resilience	CRISPRa + Base editing + Screening	- Overexpress HSPs/antioxidants. - Engineer membrane desaturases (cold tolerance). - Genome-wide CRISPRi screens for stress regulators.	Improved thermotolerance; reduced ROS; identification of novel resilience genes.	[62,198,227,228,229,230,231,232]
Carbon Capture and Utilization	Base editing + Multiplexed CRISPRi + Logic gates	- Engineer Rubisco. - Repress TCA cycle; activate lipid synthesis under N-limitation. - Integrate xylose utilization pathways.	Improved carbon flux to products; broadened substrate utilization.	[9,101,180,193,215,233,234,235]
Integrated Industrial Strain	All tools (epigenome + PE + CRISPRa/i + Base editing)	- Example: dCas9-p300 stabilizes lipid genes; PE integrates Rubisco; sHSP ↑ thermotolerance.	Multi-trait industrial strain: high lipid yield, stable expression, stress resilience.	[28,30,57,122,193,228,236,237,238,239]

**Table 3:** Comparative performance and requirements of core CRISPR tools across model and representative non-model microalgae.

Species	Model Status	Preferred Cas Variant(s)	Typical Editing Efficiency (KO)	HDR Efficiency	Major Species-Specific Challenges	Ref.
*Chlamydomonas reinhardtii* (Green Alga)	Model	SpCas9, FnCas12a	Moderate–High (10–50%)	Low (1–5%)	Low HDR, silencing, need for cell wall-deficient strains	[62,247,248,249]
*Nannochloropsis* spp. (Eustigmatophyte)	Non-model	FnCas12a, LbCas12a	High (20–80%)	Very Low (<1%)	Very low HDR, rigid cell wall, silencing, PEG sensitivity	[219,245,250,251,252,253]
*Phaeodactylum tricornutum* (Diatom)	Non-model	FnCas12a, LbCas12a	High (30–70%)	Very Low (<1%)	Very low HDR, silica cell wall, epigenetic silencing	[62,88,250,254,255,256,257,258]
*Haematococcus pluvialis* (Green Alga)	Non-model	SpCas9 (optimized)	Moderate (reported)	Very Low (<1%)	Extremely thick cell wall, low transformation efficiency, astaxanthin interference	[28,259,260,261,262,263,264]

## Data Availability

Not applicable.
